# How do differences in Achilles’ tendon moment arm lengths affect muscle-tendon dynamics and energy cost during running?

**DOI:** 10.3389/fspor.2023.1125095

**Published:** 2023-04-17

**Authors:** Eric C. Bennett, Esthevan Machado, Jared R. Fletcher

**Affiliations:** Department of Health and Physical Education, Mount Royal University, Calgary, AB, Canada

**Keywords:** strain energy storage, energy cost, running economy, ultrasound, metabolic cost of force production

## Abstract

**Introduction:**

The relationship between the Achilles tendon moment arm length (AT_MA_) and the energy cost of running (E_run_) has been disputed. Some studies suggest a short AT_MA_ reduces E_run_ while others claim a long AT_MA_ reduces E_run_. For a given ankle joint moment, a short AT_MA_ permits a higher tendon strain energy storage, whereas a long AT_MA_ reduces muscle fascicle force and muscle energy cost but shortening velocity is increased, elevating the metabolic cost. These are all conflicting mechanisms to reduce E_run_, since AT energy storage comes at a metabolic cost. Neither of these proposed mechanisms have been examined together.

**Methods:**

We measured AT_MA_ using the tendon travel method in 17 males and 3 females (24 ± 3 years, 75 ± 11 kg, 177 ± 7 cm). They ran on a motorized treadmill for 10 min at 2.5 m · s^−1^ while E_run_ was measured. AT strain energy storage, muscle lengths, velocities and muscle energy cost were calculated during time-normalized stance from force and ultrasound data. A short (SHORT *n* = 11, AT_MA_ = 29.5 ± 2.0 mm) and long (LONG, *n* = 9, AT_MA_ = 36.6 ± 2.5 mm) AT_MA_ group was considered based on a bimodal distribution of measured AT_MA._

**Results:**

Mean E_run_ was 4.9 ± 0.4 J · kg^−1^ · m^−1^. The relationship between AT_MA_ and E_run_ was not significant (*r*^2^ = 0.13, *p* = 0.12). Maximum AT force during stance was significantly lower in LONG (5,819 ± 1,202 N) compared to SHORT (6,990 ± 920 N, *p* = 0.028). Neither AT stretch nor AT strain energy storage was different between groups (mean difference: 0.3 ± 1 J · step^−1^, *p* = 0.84). Fascicle force was significantly higher in SHORT (508 ± 93 N) compared to LONG (468 ± 84 N. *p* = 0.02). Fascicle lengths and velocities were similar between groups (*p* > 0.72). Muscle energy cost was significantly lower in LONG (0.028 ± 0.08 J · kg · step^−1^) compared to SHORT (0.045 ± 0.14 J · kg · step^−1^ *p* = 0.004). There was a significant negative relationship between AT_MA_ and total muscle energy cost relative to body mass across the stance phase (*r* = −0.699, *p* < 0.001).

**Discussion:**

Together these results suggest that a LONG AT_MA_ serves to potentially reduce E_run_ by reducing the muscle energy cost of the plantarflexors during stance. The relative importance of AT energy storage and return in reducing E_run_ should be re-considered.

## Introduction

The role of the long Achilles tendon in reducing the metabolic cost of locomotion is well-established (1–5). During locomotion, the triceps surae muscles produce longitudinal forces that are transferred through the Achilles tendon, producing a joint moment. The required muscle force to achieve a given joint moment is dependent on the moment arm length. The Achilles tendon moment arm (AT_MA_) can be defined as the perpendicular distance from the centre of rotation of the ankle joint to the line of action on the Achilles tendon (6).

Generating muscle forces during stance comes at a metabolic cost, typically considered to be proportional to the rate and magnitude of muscle force generation (7). Thus, generating low forces at a low shortening velocity should come at a low metabolic cost compared to higher muscle forces and/or higher shortening velocities. As velocity increases, recruitment must increase to maintain the required force (8–10), and it is acknowledged that force, not power is the determining factor for muscle activation during running. For a given force requirement, the level of activation and therefore the energy cost, can be minimized if the muscle can operate at a slower shortening velocity (9, 10).

With regards then to the AT_MA_, for a given joint moment, a longer AT_MA_ should reduce muscle forces and thus metabolic cost. Whereas for a given joint angular rotation, a short AT_MA_ would result in a lower shortening velocity, also reducing metabolic cost. Considering these potential mechanisms, it is perhaps no surprise that the relationship between AT_MA_ and the energy cost of running (E_run_) has been contentious. Some studies have shown that a short AT_MA_ is associated with a lower oxygen cost during running (14, 15). The proposed mechanisms for the lower oxygen cost of running are presumed to be two-fold. First, for a given plantarflexion moment, a short AT_MA_ allows for a greater elastic strain energy storage and return from the AT which are recovered as kinetic energy during the stance phase (14). The AT stores elastic strain energy as it stretches, and releases a large portion of this mechanical energy as it recoils; a shorter AT_MA_ is related to a larger AT strain energy storage and return (16), which further supports previous findings suggesting a short AT_MA_ is associated with a low E_run_ (14, 15). Additionally, a short AT_MA_ allows for a lower muscle fascicle shortening and lower shortening velocity for a given joint rotation during stance, which in turn reduces metabolic cost of activating a greater volume of muscle (17–19) and muscle energy cost because of the muscle’s force-velocity relationship (9).

What has not yet been considered in these proposed explanations for the energetic benefits of a short AT_MA_ is that elastic strain energy storage does not come without a metabolic cost itself (20, 21). Indeed, the additional elastic strain energy storage associated with a short AT_MA_ is a result of higher muscle forces for a given joint moment required to stretch the Achilles tendon. Generating these higher muscle forces comes at a higher muscle energy cost (7, 22, 23). To support this notion, we recently demonstrated that the muscle energy cost was considerably higher than the mechanical energy stored and returned from the Achilles tendon during running (20), suggesting the role that the Achilles tendon plays in reducing metabolic cost may be different than previously thought. More recently, Schroeder and Kuo (21) suggested that active positive muscle mechanical work must be performed to restore dissipative energy losses during each stride, associated with the loss of the body’s centre of mass momentum when the leg collides with the ground, as well as hysteresis energy losses. We propose that the AT serves to reduce metabolic cost by decoupling the length change of muscle fascicles from the entire muscle-tendon unit, thereby reducing the metabolic cost associated with producing additional amounts of positive and/or negative work during the stance phase of running (21, 24–26).

In contrast, a long AT_MA_ has also been associated with a reduced E_run_ (27). The proposed mechanism suggests that a longer AT_MA_ allows for lower muscle forces to produce a given joint moment. The lower muscle forces reduce the muscle metabolic cost and should translate into a reduced E_run_. However, fascicle shortening and shortening velocity is increased for a given joint rotation during stance, which may increase the muscle metabolic cost and the E_run_ because a higher active muscle volume will be required as a result of the force-velocity relationship (9). However, runners with a long AT_MA_ were found to have less ankle joint rotation during stance (27), contributing to a lower triceps surae shortening velocity, and reduced active muscle volume (28), contributing to their lower E_run_ associated with a longer AT_MA_.

Studies have also been conducted on Kenyan runners, a population of runners known for their exceptional running economy (15, 29, 30). Previous studies have investigated the role of the muscle-tendon unit and foot architecture in these runners as a potential explanation for their phenomenal running economy (31–33). For example, Kunimasa et al. (31) showed that Kenyan runners have longer AT_MA_ compared to their Japanese counterparts, as well as a lower foot lever ratio, the ratio of the ground reaction force lever arm (often assumed from the forefoot length) to the AT_MA_ which appears to have persisted since birth (32), providing biomechanical and metabolic benefits since a young age (29, 34). It is important also to consider the foot lever ratio. Potential differences in foot lever ratio, may also be a potentially confounding factor in the relationship between E_run_ and AT_MA_, since a lower foot lever ratio reduces the required plantarflexion joint moment produced by the muscles during the stance phase. This potentially confounding factor has often been ignored in previously-reported E_run_ vs. AT_MA_ relationships (6, 14, 15). Could this AT_MA_ debate be settled simply because those runners with short AT_MA_ also have short forefoot lengths?

Taken together, the present literature suggests E_run_ can be reduced by two independent, and contrary mechanisms: reducing metabolic cost by storing and returning a greater amount of mechanical energy in the Achilles tendon or reducing the metabolic cost of contraction as a result of the muscle(s)’ force-length-velocity relationships. The debate may be settled if muscle energy cost and strain energy storage/return was measured during submaximal running in a cohort of runners whose AT_MA_’s differed. To date, these mechanisms have not been measured simultaneously to explain the potential role of the AT_MA_ to reducing E_run_.

The measurement of muscle fascicle and Achilles tendon length change and velocity of the triceps surae is easily performed during running (35–39). From these measurements, combined with an estimate of muscle-tendon forces using inverse dynamics (40), AT energy storage and return can be quantified and the muscle energy cost of contraction during stance can be calculated (20). The main determinant of whole-body E_run_ is the generation and maintenance of muscular force, to support and accelerate the body (7), which is influenced by the AT_MA_. The level of muscle activation (and therefore muscle volume) necessary to generate this force is dictated by the muscle’s force-length-velocity relationship (41–43). A logical mechanism for a reduced E_run_ with either short or long AT_MA_ should be demonstrated by potential differences in the muscle’s length and velocity during stance relative to the muscle’s force-length and force-velocity potentials (43).

Therefore, the primary purpose of this study was to quantify AT energy storage and return and the muscle metabolic cost during submaximal running in runners who possessed a range of AT_MA_. A secondary purpose was to explain running energetics from a force-length-velocity relationship perspective and if differences in AT_MA_ affect these fundamental skeletal muscle properties during running. Together, these results may offer insight into differences in muscle-tendon dynamics across AT_MA_ lengths.

## Methods

### Participant characteristics

20 healthy, recreationally active participants (17 males, 3 females, 24 ± 3 years, 75 ± 11 kg, 177 ± 7 cm) completed the experimental protocol. The participants were recreationally-trained runners. The inclusion criteria were that the participants were between 18 and 50 years old, could achieve a steady-state in oxygen uptake (V̇O_2_) during a 10-min run at the required speed of 2.5 m · s^−1^, and had no lower leg injuries within the last 6 months. We aimed to recruit a diverse group of participants to have a wide range of both E_run_ and AT_MA_. The participants gave their informed written consent to participate in the experimental protocol which was approved by the Mount Royal University Human Research Ethics Board (HREB ID #102674).

### Experimental protocol

The participants visited the lab on a single occasion. Each participant’s AT_MA_ was estimated using the tendon excursion method, accounting for passive forces (44). The participants laid prone on a dynamometer (Biodex Medical Systems Inc., Shirley, NY, USA) with their right knee fully extended. The shank and unshod right foot were affixed to the dynamometer using Velcro straps, with the ankle at 90°. Ankle angle was defined as the angle of the foot relative to the long axis of the shank. Briefly, the ankle was passively rotated at 0.1745 rad · s^−1^ through the participant’s voluntary range of motion. A 12.5 MHz linear array B-mode ultrasound probe (65 mm, LV8-4L65S-3, MicrUS EXT-1H, Telemed, Vilnius, Lithuania) was used to visualize the medial gastrocnemius (MG) myotendinous junction (MTJ). Ultrasound images were recorded at 39 Hz. The displacement of the MTJ was tracked from 85° to 95°, using *ImageJ* (v.2.3.0/1.53s, NIH, Baltimore MD USA). AT moment arm was calculated as the ratio of MTJ displacement (in mm) to ankle joint rotation (in radians). The bias and limits of agreement compared to the caliper method for the tendon travel method, previously reported by Fletcher and MacIntosh (44) are 0.1 and 1.5 mm, respectively. The intraclass correlation coefficient for test-retest reliability has previously been reported to be *r* = 0.88 (44).

Since the inter-individual variation in body height was large, and a significant relationship was seen between AT_MA_ and body height (see results), AT_MA_ was normalized to body height as described previously by Scholz et al. (14) and we present both absolute and height-normalized AT_MA_ where appropriate.

Following this, participants ran at a speed of 2.5 m · s^−1^ on a motorized treadmill (Woodway Pro, Woodway USA, Waukeshka, WA) for 10 min. During the run, expired V̇O_2_ and V̇CO_2_ were measured to quantify E_run_ using a metabolic cart (Quark CPET, Cosmed, Rome, Italy) according to Fletcher et al. (45) and expressed an energy cost (J · kg^−1^ · m^−1^) as it is a more sensitive and reliable assessment of running economy compared to the measurement of steady-state V̇O_2_ alone (46–48). Prior to each testing session, the metabolic cart was calibrated using room air and a gas mixture of known composition (5% CO_2_% and 16% O_2_). The flow sensor was calibrated manually with a 3l syringe. Muscle-fascicle dynamics were calculated from ultrasonography and inverse dynamics during the last minute of the 10-min run, and the middle 10 consecutive steps were identified and used for further analyses. Expired gases were collected for the entire duration of the run. All participants achieved a steady-state V̇O_2_ (defined as a change of <200 ml/min for any 15s period during the last 2 min of the run. The average V̇O_2_ and V̇CO_2_ over the last 3 min were used to calculate E_run_.

E_run_ (J · kg^−1^ · m^−1^) was calculated from the average V̇O_2_ and V̇CO_2_ over the final 3 min of the run from the metabolic equation presented by Peronnet and Massicotte (49), which expresses the rate of energy expenditure in kJ · s^−1^. We then expressed this rate of energy expenditure as a relative energy cost per unit distance *(J · kg^−1^ · m^−1^)*:

$$E_{run}(J\cdot kg^{-1}\cdot m^{-1})=16.89\dot{V}O_{2}+4.84\dot{V}CO_{2}xBM^{-1}x\;s^{-1}\times 1000$$

where *V˙O_2_* and *V˙CO_2_* is in L · s^−1^, *BM* is body mass (in kg), *s* is speed (in m · s^−1^) and 1,000 J · kJ^−1^

### Muscle fascicle length change

The MG muscle fascicle of the right leg was imaged using a second 12.5 MHz linear array B-mode ultrasound probe (60 mm, LV8-5N60-A2, ArtUs EXT-1H, Telemed, Vilnius, Lithuania) at a sampling frequency of 70 Hz. The MG muscle was chosen over other triceps surae muscles because Lai et al. (37) showed that MG muscle fascicle length changes during the stance phase of running were the largest of the triceps surae muscles.

The MG fascicle lengths and MTJ shortening/elongation were measured manually using *ImageJ* from the respective ultrasound images during the stance phase. Fascicle and AT length change, velocity, work and power were calculated at each 5% of stance for the entire stance phase and averaged over 10 consecutive stance phases. To correct for AT shortening and lengthening as a result of changes in ankle joint angle, ankle angle was measured using a high-speed video camera (Ziqian, N5 1080p Webcam, 50 Hz). Ankle angle was measured at each instance during stance using *Tracker* (v. 6.0.8, Open Source Physics, Compadre.org/osp). AT length change due to ankle joint rotation (in mm) during the stance phase was calculated from the ankle joint change (in radians) and the measured AT moment arm length (in mm), a derivation of the equation to calculate AT moment arm from the tendon excursion method (44, 50). Instantaneous tendon length was estimated by subtracting the measured MG fascicle length, taking the effect of muscle pennation angle into account (51).

### Kinematics and kinetics

Vertical ground reaction forces of the right foot were measured using a commercially available instrumented insole (Loadsol, Novel.de, St Paul MN USA), collected at 100 Hz during the last minute of the run. These insoles have been shown to be reliable and valid compared to inverse dynamics, at a significantly reduced cost. Specifically, Hullfish and Baxter (40) showed peak plantarflexion moment to be on average 5.4% higher using the insoles compared to inverse dynamics using marker-based motion capture and a force-measuring treadmill; however, the 95% CI for the difference between the two measurements included 0% difference. Data were saved to a smart device (iPad mini-4, Apple Inc. Cupertino CA) for subsequently analyzed according to Hullfish and Baxter (40).

Plantarflexion moment during the stance phase was calculated according to Hullfish and Baxter (40). The force insole has three force sensing zones, which we treated as discrete one-dimensional force plates, assuming the measured ground reaction forces were orthogonally directed and in the middle of each force-sensing zone. The geometric centres of pressure of each force-sensing zone were measured using digital calipers (Mastercraft Tool Co, Earth City, MO) to the nearest 0.02 mm. the reported accuracy of the calipers. The moment arms of each zones were then calculated by subtracting the distance between the posterior sensor and the ankle joint. We then calculated sagittal plane plantar flexion moment as the sum of the products of each zone moment arm and the applied load (40). AT force was calculated from the calculated plantarflexion moment divided by the measured AT_MA_. MG force was estimated based on the relative physiological cross-sectional area of all ankle plantarflexors (0.1746, 52) divided by the cosine of the measured pennation angle of the MG muscle fascicle. The foot lever ratio was determined as the length of the forefoot, divided by the AT_MA_ length (31). Mechanical work performed by the MG and AT, respectively, was calculated as the integral of fascicle (or tendon) force and length change over the entire stance phase. Positive fascicle work was considered fascicle shortening. AT positive work, a measure of AT strain energy storage, was calculated by integrating the AT force over the measured AT elongation, omitting elongations below the length at heelstrike, in order to quantify AT energy storage/return during the stance phase alone (39).

### Electromyography

Three wireless electromyography (EMG) sensors (Delsys Trigno, Natick Massachusetts, USA) were placed on the participants’ right lower leg, using double-sided stickers, along the presumed fascicle angle according to SENIAM guidelines (53). The sensors have four 1 mm x 5 mm parallel bars (contacts), of 99.9% silver with a fixed inter-electrode distance of 10 mm. These sensors were located on the lateral gastrocnemius (LG), soleus (SOL), and tibialis anterior (TA). EMG signals were collected at 2,048 Hz during the last 2 min of each trial. To reduce noise and signal artifact, the signal was filtered through a 5th order Butterworth filter (high and low pass filter of 20 and 500 Hz, respectively). EMG amplitude was calculated as the root mean square (RMS) of the raw, filtered EMG signal. This RMS was interpreted as the level of muscle activation during stance: a combination of motor unit recruitment and rate coding.

### Muscle energy cost

In order to compare the metabolic energy cost required to store elastic strain energy within the AT, the MG energy cost was calculated over the entire stance phase according to Fletcher and MacIntosh (20), which has been described in detail elsewhere (20, 54, 55). In brief, the metabolic cost of the MG during the stance phase was determined from the estimated number of in-parallel crossbridges that were needed to generate the measured MG force, the amount of crossbridge cycles to accommodate MG fascicle shortening and the amount of half-sarcomeres in series from the measured MG fascicle length. The estimated number of in-parallel crossbridges was derived from the MG force divided by the estimated force per crossbridge. The force per crossbridge decreases with increasing shortening velocity from a crossbridge force of 3 pN under near-isometric conditions (53) to 0 pN at maximal shortening velocity based on the linear sarcomere force-velocity relationship (57). Sarcomere shortening velocity (*V*) was calculated from the instantaneously measured fascicle shortening velocity throughout the stance phase and scaled to maximal shortening velocity (*V_max_*). We assumed a maximal fascicle shortening velocity of 10.6 fascicle lengths · s^−1^ which was calculated from the assumed maximal shortening velocities of Type I and Type II fibers of 4.4 fascicle lengths · s^−1^and 16.8 fascicle lengths · s^−1^ at physiological temperatures (38) and assuming the MG consisted of 50% Type I fibres (58). We expressed total muscle energy cost across the entire stance phase relative to body mass since E_run_ is determined primarily by the energy needed for muscle contraction of sufficient average force to support body weight for the full stride duration (7). Therefore, average muscle force and thus muscle energy cost is related to the average vertical force (Fz) during stance, as dictated by body mass and running speed and the Fz moment arm and the moment arm of the Achilles tendon (59, 60).

### Force-length-velocity relationships

The muscle fascicle operating range on the force-velocity relationship (61), scaled to activation (8), was estimated from the measured fascicle shortening velocity (*v*) relative to maximal shortening velocity (*V_max_* of 10.6 Lf · s^−1^). The measured muscle fascicle force (*P*) was scaled to the maximal isometric force (*P_o_*), the latter of which was measured during a maximal isometric voluntary contraction at 90° ankle angle, which is considered the short-side of the plateau region of the MG’s force-length relationship (62). The operating range of the muscle fascicles on the force-length relationship was assessed from the calculated fascicle force relative to maximum (*P/P_o_*) and the estimated sarcomere length during stance. Estimated sarcomere length was calculated assuming a sarcomere length of 2.6 µm at the short side of the plateau region of the sarcomere force-length relationship (63). Sarcomere lengths during stance (*L*) were then estimated from the measured fascicle length change relative to the fascicle length measured during the maximal isometric contraction. This fascicle length was considered maximal optimal length (*L_o_*) of 2.6 µm. Thus, sarcomere lengths during stance (*L*) could be calculated as the relative change in fascicle length at *L_o_* to the measured fascicle length during stance since length change must be accommodated from changes in sarcomere lengths.

The number of half-sarcomeres in series was determined as the ratio of the measured fascicle length (in µm) to sarcomere length, assuming an optimal sarcomere length at maximal activation of 2.6 µm (63). Thus, this method allowed us to convert the measured fascicle lengths (in mm) to estimated sarcomere lengths (in µm). The number of crossbridge cycles during stance was determined from the measured MG fascicle length change during stance. We assumed that for each crossbridge cycle the filaments move 10 nm (64). We worked under the assumption that for each crossbridge cycle, one adenosine triphosphate (ATP) was consumed (65), and for each mol of ATP, 48 kJ of energy was released per mol ATP consumed (66).

### Statistics

Values are presented as mean ± standard deviation unless otherwise indicated. We classified individuals having “SHORT” (*n* = 11, 29.5 ± 1.9 mm) or “LONG” (*n* = 9, 36.6 ± 2.5 mm) AT_MA_ based on a bimodal distribution of AT_MA_. Statistical analysis was performed using *JASP* (Version 0.16.2.0). Shapiro-Wilk tests were performed to test for normality and Levene’s tested for equality of variance of all dependent variables. Student Independent samples *t*-tests were utilized to determine differences between AT_MA_, E_run_, stance time and foot lever ratio between groups. Two-way repeated measures analysis of variance (ANOVA) for unequal sample sizes between groups (with Type III sum of squares to adjust for unequal sample sizes) was used to test effects of group on the dependent variables. A two-way repeated-measures ANOVA was used to test for differences in MG muscle energy cost at every 5% interval across the stance phase, with %stance as the repeated measures factor and group as the between subject factor. Similarly, three-way repeated measures ANOVAs for unequal sample sizes between groups (with adjusted Type III sum of squares) were also performed to test for differences in force-length (force × length × group) and force-velocity (force × velocity × group) relations between groups. Tukey’s *post-hoc* tests were used to detect significant differences between groups during stance based on dependent variables when there was no significant interaction but a significant simple main effect of group. The effect sizes were determined using Cohen’s *d*, with small, medium, and large sizes being *d* ≥ 0.2, *d* ≥ 0.5, *d* ≥ 0.8, respectively. The *a priori* level of significance was set at *p* < 0.05.

## Results

AT_MA_ for all participants was 32.7 ± 4.2 mm. AT_MA_ was 29.5 ± 1.9 mm in SHORT (*n* = 11) and 36.6 ± 2.5 mm for LONG (*n* = 9, *p* < 0.001). Height and weight were significantly greater in LONG (181.4 ± 3.5 cm, 81.8 ± 8.5 kg) compared to short (173.8 ± 7.3 cm, 70.2 ± 10.9 kg, *p* = 0.011 and *p* = 0.019, respectively). AT_MA_ length was also significantly positively correlated with both height (*r*^2^ = 0.228, *p* = 0.048) and weight (*r*^2^ = 0.214, *p* = 0.04). Height-normalized AT_MA_ was also significantly greater in LONG (0.020 ± 0.002) compared to SHORT (0.017 ± 0.001, *p* = 0.0002).

**Figure 1:**
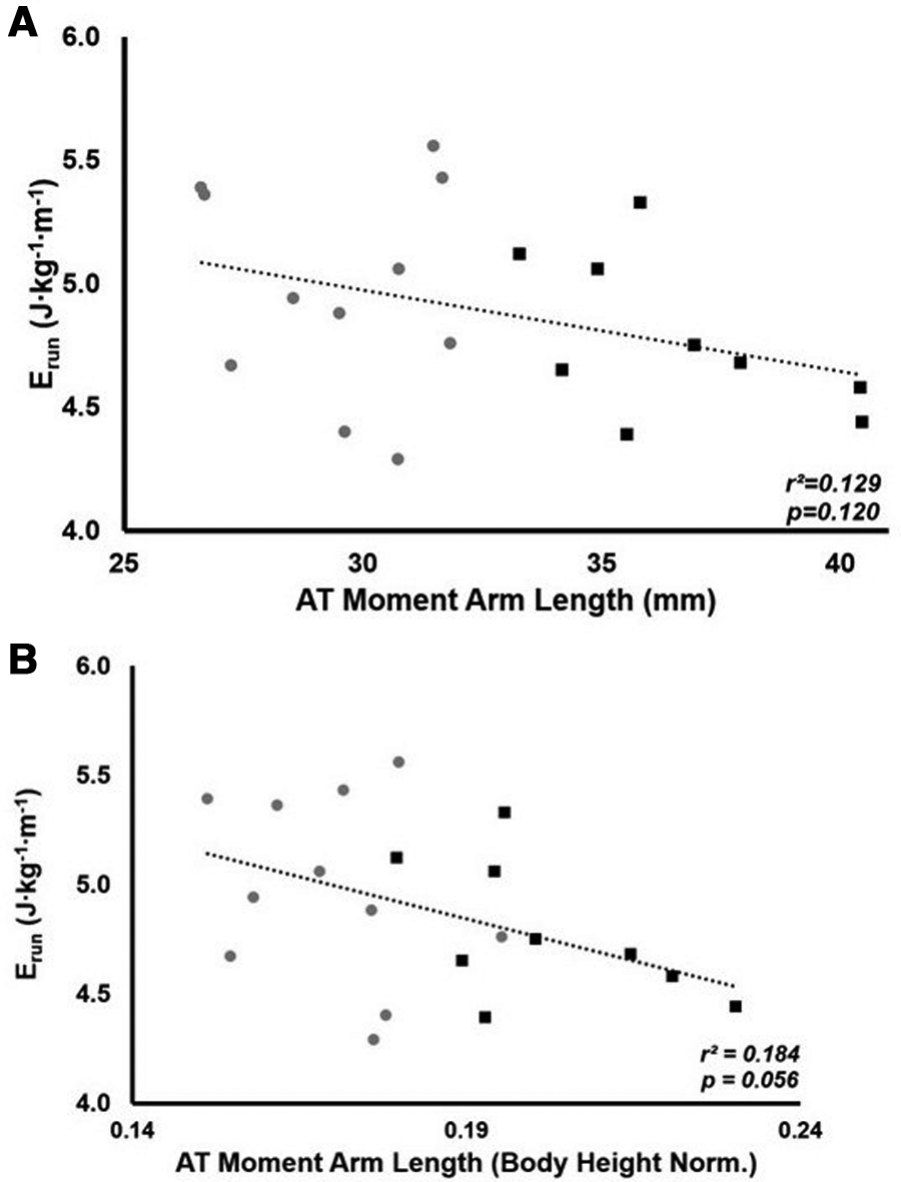
The relationship between the energy cost of running (E_run_) and AT_MA_ (**A**) and the relationship between E_run_ and height normalized AT_MA_ (**B**). Grey circles represent the SHORT AT_MA_ group, while black squares represent the LONG AT_MA_ group.

The mean E_run_ for all participants was 4.89 ± 0.39 J · kg^−1^ · m^−1^. There was no significant relationship between AT_MA_ and E_run_ (*r^2^* = 0.129, *p* = 0.120, Figure 1A). There was also no significant group difference in E_run_ (LONG 4.78 ± 0.32 vs. SHORT 4.98 ± 0.43 J · kg^−1^ · m^−1^, *p* = 0.265); however, a medium effect size for E_run_ was seen (*d* = 0.53). When AT_MA_ was normalized to height, a negative relationship between AT_MA_ and E_run_ was demonstrated; however, this relationship was not significant (*r*^2^ = 0.184, *p* = 0.056, Figure 1B).

**Figure 2:**
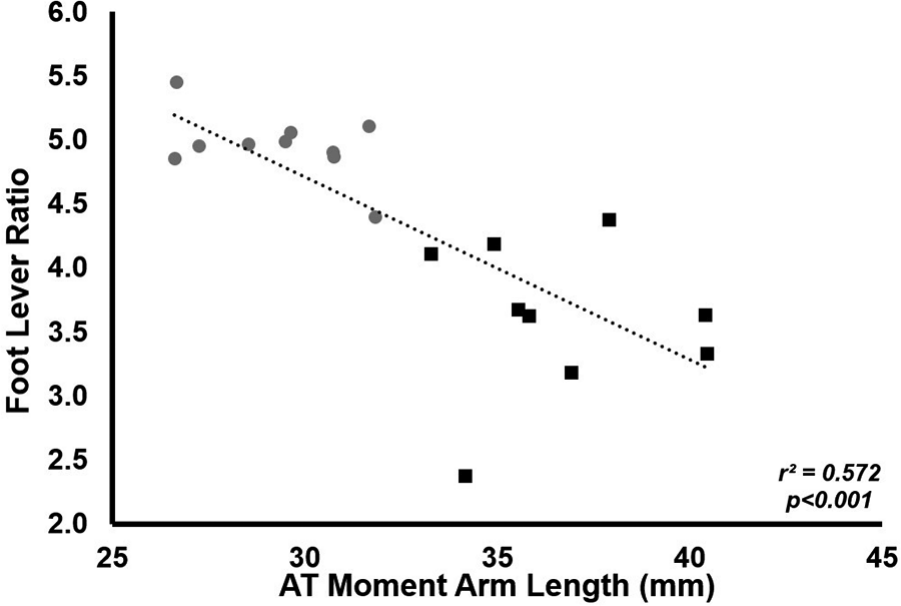
The relationship between foot lever ratio and AT_MA_. Grey circles represent the SHORT AT_MA_ group, while black squares represent the LONG AT_MA_ group. Across all participants, a longer AT_MA_ was associated with a lower foot lever ratio.

Neither stance time (LONG 0.347 ± 0.044 ms vs. SHORT 0.346 ± 0.025 ms, *p* = 0.985), ankle joint excursion (49 ± 4° for LONG, 50 ± 5° for SHORT, *p* = 0.506) plantarflexion moment (207 ± 35 Nm for LONG, 204 ± 30 Nm for SHORT, *p* = 0.979) or average ground reaction force lever arm (132 ± 23 mm for LONG, 145 ± 9 mm for SHORT, *p* = 0.159) was significantly different between groups. Foot lever ratio was lower in LONG (3.6 ± 0.6) compared to SHORT (5.0 ± 0.3, *p* < 0.0001). Foot lever ratio was negatively correlated with AT_MA_ (*r*^2^ = 0.572, *p* < 0.001,) with LONG having a smaller foot lever ratio (Figure 2).

**Figure 3:**
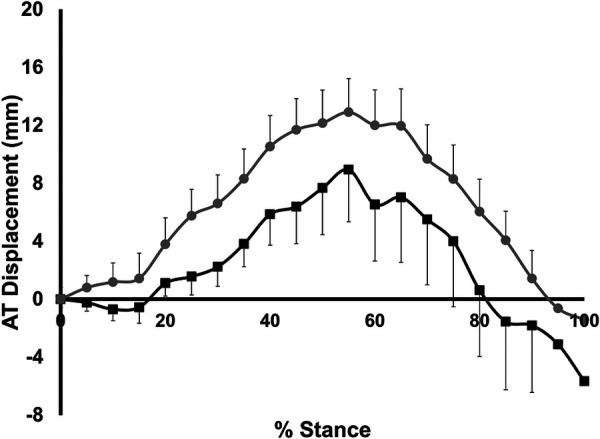
Time-normalized AT displacement over the stance phase of running relative to the AT length measured at heelstrike. Values are presented as mean ± SD. Grey circles represent the SHORT AT_MA_ group, while black squares represent the LONG AT_MA_ group.

AT stretch and recoil is shown in Figure 3. For both groups, the AT was stretched until 55% of stance, and then recoiled until toe-off (ie. 100% of stance). Maximum AT stretch during stance was not different between groups (10.3 ± 7.2 mm for LONG, 14.0 ± 6.7 mm for SHORT, *p* = 0.264). There was no significant group x stance interaction for AT stretch or recoil (*p* = 0.968) nor a significant main effect of group (*p* = 0.169).

**Figure 4:**
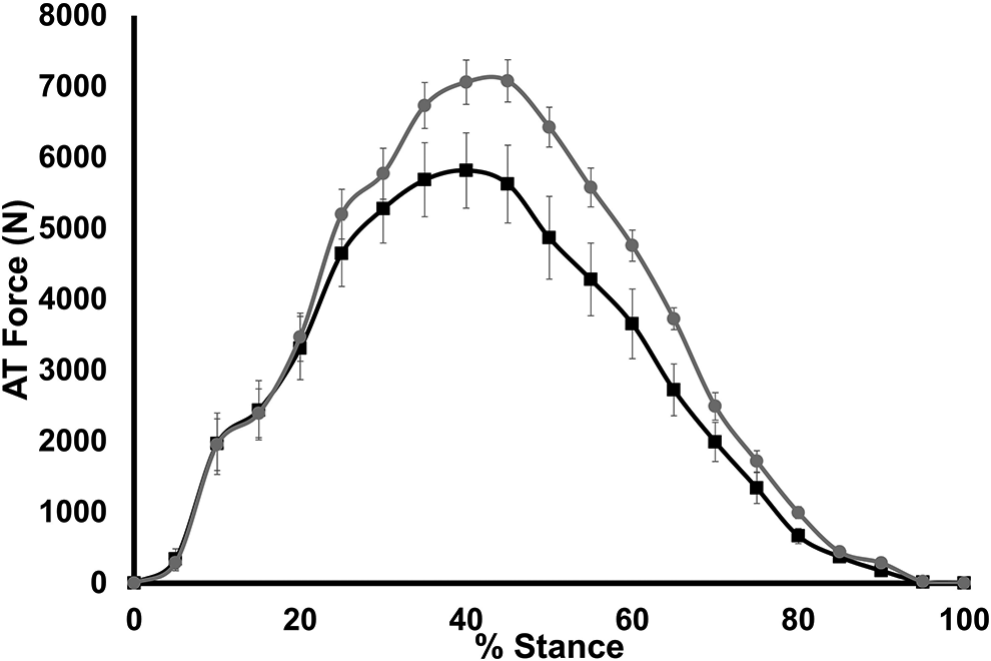
Time-normalized AT force over the stance phase of running. Values are presented as mean ± SD. Grey circles represent the SHORT AT_MA_ group, while black squares represent the LONG AT_MA_ group.

AT force measured during stance is shown in Figure 4. A significant group × stance interaction was found for AT force (*p* < 0.001); however, a significant main effect of group for AT force was not seen (*p* = 0.07). Maximum AT force during stance was significantly lower in LONG (5,819 ± 1,202 N) compared to SHORT (6,990 ± 920 N, *p* = 0.028).

**Figure 5:**
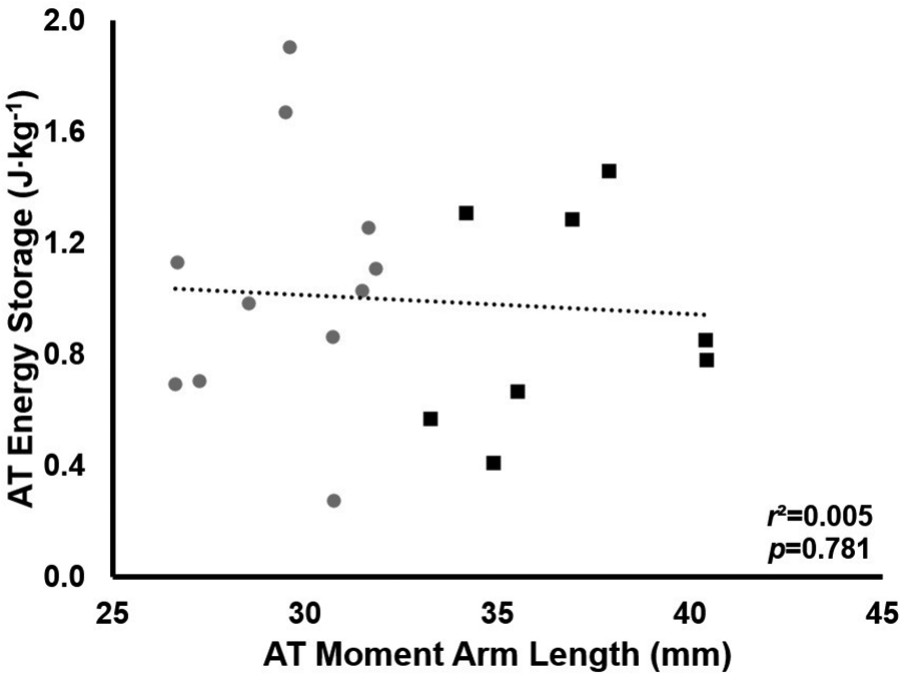
The relationship between total AT energy storage and AT_MA_. Grey circles represent the SHORT AT_MA_ group, while black squares represent the LONG AT_MA_ group. No relationship between AT_MA_ and total AT energy storage was found.

Maximal AT strain energy storage was 16 ± 6 J · step^−1^ in LONG and 15 ± 5 J · step^−1^ in SHORT (mean difference across the stance phase: 0.3 ± 1 J · step^−1^, *p* = 0.84). Total strain energy storage during stance was also not different between groups (165 ± 24 J · step-1 in SHORT vs. 182 ± 75 J · step-1 in LONG, *p* = 0.63). AT strain energy storage was also not significantly correlated with AT_MA_ (*r*^2^ = 0.005, *p* = 0.781, Figure 5).

There was no significant group x stance interaction for AT velocity as a function of stance (*p* = 0.29), nor a significant main effect of group for AT velocity (*p* = 0.338). There was also no significant group x stance interaction nor a main effect of group for AT power during stance (*p* = 0.748).

**Figure 6:**
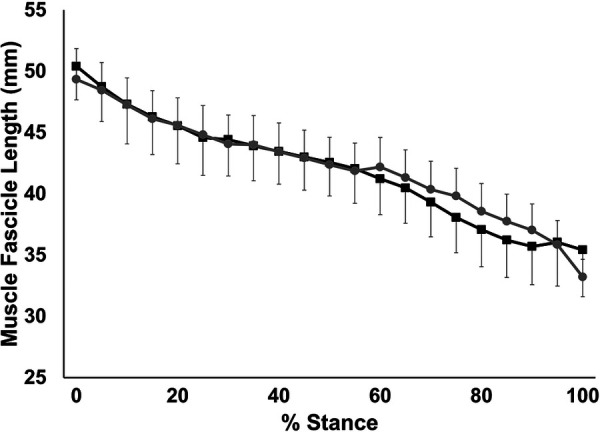
Time-normalized fascicle length over the stance phase of running. Values are presented as mean ± SD. Grey circles represent the SHORT AT_MA_ group, while black squares represent the LONG AT_MA_ group.

Muscle fascicle shortening during stance is shown in Figure 6. Muscle fascicles shortened continuously throughout stance (*p* < 0.001). There was no significant group x stance interaction (*p* = 0.988) nor a significant main effect of group (*p* = 0.95). Similarly, no significant group × stance interaction, nor a significant main effect of group was seen for fascicle shortening velocity (*p* > 0.717) nor fascicle work during stance (*p* > 0.943).

**Figure 7:**
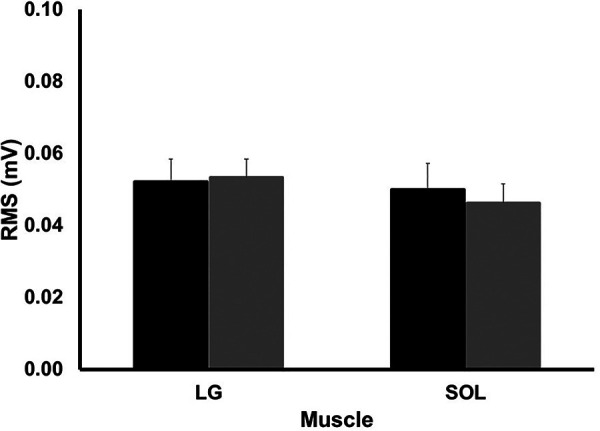
EMG amplitude for lateral gastrocnemius (LG) and soleus (SOL) during the stance phase of running. Values are presented as mean ± SD. Black bars represent the long AT_MA_ group while the grey bars represent the short AT_MA_ group.

The magnitude of muscle activation, assessed by EMG, was not different between groups for either LG or SOL (*p* > 0.562). This is shown in Figure 7.

**Figure 8:**
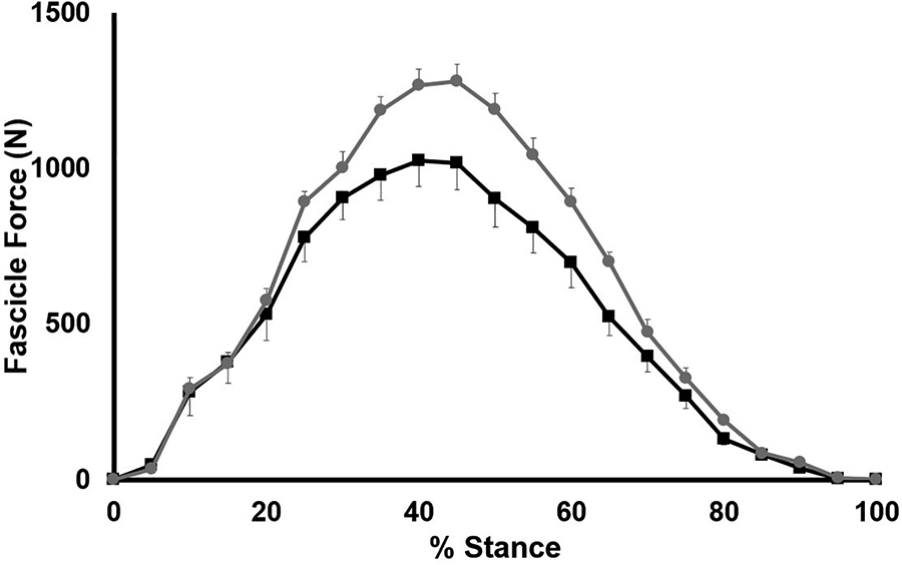
Time-normalized AT force over the stance phase of running. Values are presented as mean ± SD. Grey circles represent the SHORT AT_MA_ group, while black squares represent the LONG AT_MA_ group.

We demonstrate a significant group x stance interaction for fascicle force (*p* < 0.001 and a significant main effect of group (*p* = 0.024); SHORT had significantly higher fascicle forces during stance than LONG (Figure 8). *Post-hoc* testing revealed a significantly lower fascicle force in LONG between 35% and 60% of stance (*p* < 0.04). Neither fascicle length change, fascicle velocity or fascicle work during stance was significantly different between groups (*p* > 0.741).

**Figure 9:**
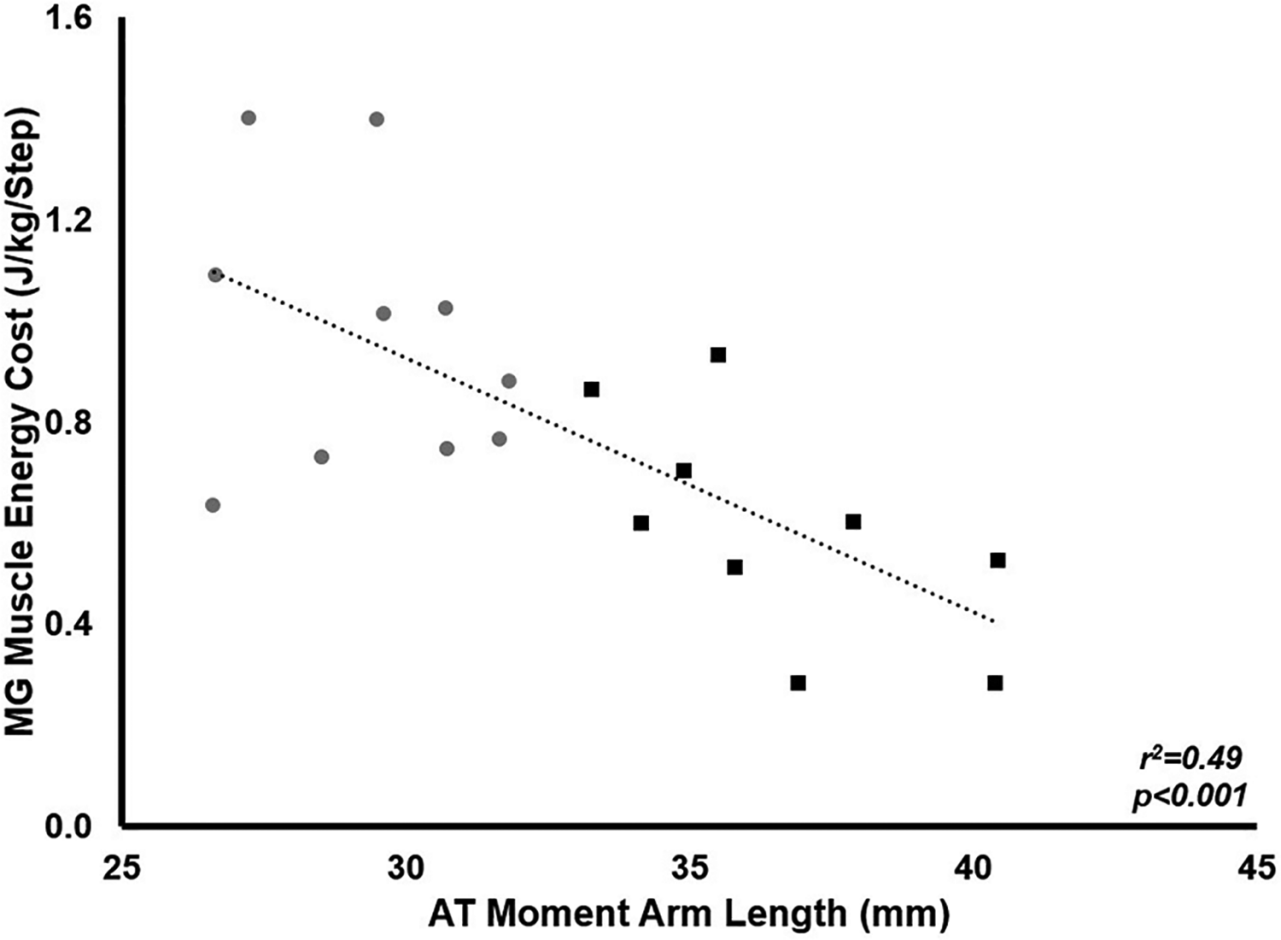
The relationship between MG muscle energy cost during stance and AT_MA_. Grey circles represent the SHORT AT_MA_ group, while black squares represent the LONG AT_MA_ group. Across all participants, a longer AT_MA_ was associated with a lower muscle energy cost during stance.

Muscle energy cost relative to body mass was significantly lower in LONG (0.028 ± 0.08 J · kg^−1^ · step^−1^) compared to SHORT (0.045 ± 0.14 J · kg^−1^ · step^−1^ *p* = 0.004). There was a significant negative relationship between AT_MA_ and total muscle energy cost across the stance phase relative to body mass across all participants (*r*^2^ = 0.49, *p* < 0.001), suggesting longer AT_MA_ were associated with a reduced mass-specific muscle energy cost during stance (Figure 9).

**Figure 10:**
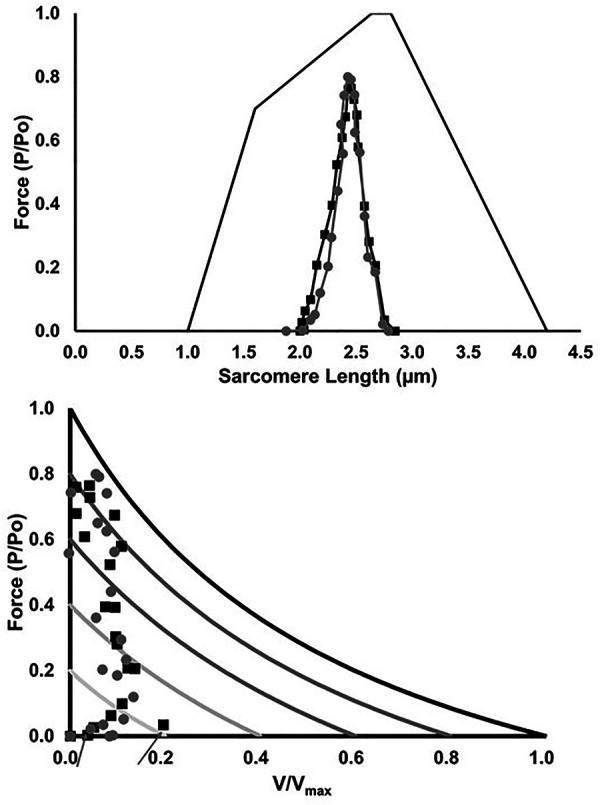
Muscle fascicle operating range on the force-length (top) and force-velocity relationship (bottom), respectively. Values are presented as mean ± SD. Grey circles represent the SHORT AT_MA_ group, while black squares represent the LONG AT_MA_ group. The force-velocity relationship is scaled to level of muscle activation. Aarows show the higher shortening velocity in LONG during the first 5% of the stance phase (*p* = 0.03) compared to SHORT.

The estimated *in vivo* operating range of the MG fascicles on the force-length and force-velocity relationships are shown in Figure 10. There was no significant main effect of group on force at a given sarcomere length (*p* > 0.17), nor any significant group differences in the estimated sarcomere length during stance (*p* > 0.64). With regards to the force-velocity relationship, similar forces and shortening velocities were seen between groups, with the exception of a higher shortening velocity in LONG during the first 5% of the stance phase (*p* = 0.03).

## Discussion

This study set out to contribute to the debated influence of AT_MA_ on reductions in E_run_. This debate stems from several contrary and unresolved observations: (1) E_run_ has been shown to be reduced with both a short AT_MA_ (6, 14, 67) and a long AT_MA_ (15, 27). Contributing to this initial debate, the results of our study show that AT_MA_ length of recreationally-trained runners was not related to E_run_; however, we acknowledge a small sample size may have precluded showing a significant relationship between AT_MA_ and E_run_. If the results presented here were sufficiently powered, we would observe a significant negative relationship between AT_MA_ and E_run_: A long AT_MA_ would result in a lower E_run_ and there are several possibilities why that may indeed be the case.

The influence of the AT_MA_ on E_run_ stems from two primary mechanisms: a short AT_MA_ increases muscle force for a given joint moment, and a greater energy storage and return of elastic strain energy during the stance phase and/or for a given joint rotation, muscle fascicles may shorten less in runners with shorter AT_MA_. On the first mechanism, Foster et al. (16) found that elastic energy storage was negatively correlated with AT_MA_. Showing that shorter AT_MA_ were associated with a lower E_run_, Scholz et al. (14) estimated that for a given AT stiffness, a 10% shorter AT_MA_ would result in an extra mechanical energy storage of ∼7.4 J · step^−1^, or an approximate mechanical power savings of 22 W (7.4 J · step^−1^ × 3 stance phases · s^−1^). Assuming a maximum muscle efficiency of 25% (68), a 22 W reduction in mechanical power would result in a metabolic power savings of 88 W, or about 8% of the total metabolic energy for someone running 16 km/hr with a steady-state V̇O_2_ of 50 ml · kg^−1^ · min^−1^. A closer examination of these data show that for at least a few participants, their “steady-state” V̇O_2_ used to determine E_run_ was greater than (participant 13) or very near (>90%, participants 10 and 11, respectively) their reported V̇O_2_max, making it very unlikely that at least these three participants achieved a steady-state V̇O_2_ during the assessment of E_run_; their E_run_ would have been underestimated due to the (likely substantial) additional anerobic energy contribution, which would not have been reflected in the measurement of V̇O_2_ (69).

The estimates of higher elastic strain energy return with short AT_MA_ also ignore the additional metabolic cost required to store this elastic strain energy (20). Indeed, an additional muscle metabolic cost would be required to generate the ∼500 N per step, as estimated from Ker (59), associated with a 10% reduction in AT_MA_ if joint moment was held constant. To confirm this notion, here we demonstrate that AT forces were indeed ∼20% higher in SHORT compared to LONG across the stance phase, and significantly greater during midstance, yet AT strain energy storage was not significantly different between groups since AT strain during stance was also not different between groups. It could be expected that if AT force was higher in SHORT, this would be accompanied by a higher EMG activity as well. While this has been demonstrated previously in runners with varying AT_MA_ (27), we were only able to demonstrate a small (Cohen’s *d* < 0.25), but non-significant effect of AT_MA_ on the level of muscle activation during stance.

We have recently argued that the metabolic cost of force generation (and muscle shortening) is substantially higher than the mechanical energy return from the AT during running (20), emphasized by the fact that highly-trained runners had the lowest AT strain energy storage/return but also the lowest metabolic cost of muscle contraction during stance (20). In the present study, we estimated this muscle energy cost relative to the AT energy storage/return during the stance phase for the first time in runners whose AT_MA_ differed. To contribute to the LONG vs. SHORT AT_MA_ debate: MG muscle energy cost relative to body mass was significantly lower in LONG compared to SHORT, and a significant negative relationship existed between AT_MA_ and total MG muscle energy cost during stance. A lower muscle energy cost during stance would reduce the whole-body E_run_ (42, 70).

The second potential mechanism for how a short AT_MA_ might reduce E_run_ is based on the measurement of AT_MA_ using the tendon travel method itself: AT_MA_ is calculated as the ratio of muscle-tendon length change for a given joint rotation. Ankle joint excursions are relatively small, and are reduced in runners with low E_run_ (68, 71). We did not see a significant difference ankle joint excursion, fascicle length change or fascicle shortening velocity during the stance phase between groups, suggesting the impact of AT_MA_ on muscle-tendon unit length change for a given joint rotation (and subsequent energy cost of muscle shortening) was negligible.

The AT_MA_ may also influence the muscle force-length-velocity relationships, since for a given joint moment, higher AT (and therefore muscle) forces are required during the stance phase. From an E_run_ perspective, higher forces at any given length would require a higher level of activation and a concomitant increase in the energy cost associated with ion transport. Similarly, the AT_MA_ would in theory influence the muscle shortening velocity. For a given joint displacement, muscle fascicle shortening would be lower in runners with a short AT_MA_ and the cost of activation would also be reduced as a result of the muscle’s force-velocity relationship. This would be countered by the requisite higher muscle forces for a given joint moment for runners with short AT_MA_. These combined effects may explain why we saw small but significant reduction in muscle energy cost in runners with long AT_MA_ compared to short AT_MA_. The energy cost of generating force is relatively higher than the cost of activation (66, 72, 73), the former being higher in runners with short AT_MA._

When comparing E_run_ across runners of different anthropometrics, the notion that short AT_MA_ may be beneficial in reducing E_run_ assumes that the vertical ground reaction force moments generated during stance are similar between runners whose AT_MA_ differ. This is only true if, during stance, the average ground reaction force lever arm is consistent across runners of different AT_MA_. We confirm the results of others suggesting the ground reaction force lever arm is different across runners of different AT_MA_. The external lever arm is largely affected by the length of the forefoot (14, 31, 32, 74). Indeed, our results support the results of Kunimasa et al. (31) who showed that Kenyans had a smaller foot lever ratio (shorter forefoot and longer AT_MA_) compared to Japanese runners. It has been previously suggested that this foot lever ratio may affect the energy cost of locomotion (60, 75) so we argue that AT_MA_ is but one of the many factors influencing the E_run_ among many anatomical and morphological properties (76, 77). Taken together, a short forefoot length and a long AT_MA_ increases the foot lever ratio such that for a given vertical ground reaction dorsiflexion moment, a lower muscle force (and subsequently lower muscle metabolic cost) would be required. In calculating the muscle metabolic cost during the stance phase from estimates of sarcomere forces and mechanics (54, 55), we show a lower mass-specific metabolic cost of the muscle in LONG compared to SHORT, primarily as a result of lower required muscle forces during the stance phase.

### Limitations

Our study is not without several limitations. We acknowledge that our results may only apply to recreationally-trained runners at one (relatively slow) running speed of 2.5 m · s^−1^. Fletcher and MacIntosh (20) show that both muscle energy cost and AT strain energy return increase with speed, and we cannot discount that the relative contribution of AT strain energy may be more important at higher running speeds than the 2.5 m · s^−1^ tested here (16); however, muscle energy cost also increases with running speed (20), since higher forces need to be generated at a faster rate, elevating the muscle and whole-body metabolic costs (7). Kovács et al. (27) also found that the relationship between AT_MA_ and E_run_ got stronger (longer AT_MA_ result in lower E_run_) with an increase in running speed, so while we cannot speculate on the impact of faster or slower running speeds on the reported AT_MA_ vs. E_run_, we would anticipate our results to be similar at faster running speeds: (1) that mechanical strain energy storage and return is lower than the muscle metabolic cost required to store that strain energy and (2) AT_MA_ is but one factor influencing the E_run_ (favorably or unfavorably).

We also chose to assess plantarflexion moments using a commercially-available insole, rather than the gold-standard inverse dynamics approach using marker-based motion capture while running on a force-plate embedded treadmill. These force insoles have recently been shown to be reliable and valid by our colleagues and are a fraction of the cost of inverse dynamics approaches (40). These authors have previously demonstrated that peak plantarflexion moment during running was 5.4% higher using these force insoles compared to the gold-standard; however the 95% CI for these data contained 0% error, suggesting the mean difference between methods was not significantly different (40). Together, we are confident that estimating muscle forces from joint moments using commercially-available insoles is both valid and reliable while being relatively low-cost and simple to implement in and outside of the laboratory.

Despite showing a significant group difference in the estimated muscle metabolic cost during the stance phase, we are unable to demonstrate significant group differences in whole-body E_run_, although a moderate effect size (*d* = 0.53) was found. Based on this effect size, we would have required a *post hoc* sample size of *n* = 46 participants per group to demonstrate a statistical power >80%. We deemed this sample size too cost and time prohibitive and thus have reported the results found in 20 recreationally-trained runners (*n* = 9 and *n* = 11 per group, respectively). Perhaps with additional participants, we would be able to show a statistically significant negative relationship between AT_MA_ and E_run_ such that longer AT_MA_ can be associated with a lower whole-body energy cost of running. We also must acknowledge that the MG is but one of the triceps surae muscles representing a small proportion of the total triceps surae physiological cross-sectional area (∼17%, 52) so even large changes in muscle energy cost may not be reflected in whole-body metabolic cost. Future research should investigate muscle-specific energy cost of other muscles (for example those crossing the knee) in runners whose AT_MA_ differ in order to strengthen our understanding of how AT_MA_ might influence E_run_ directly.

Lastly, we must acknowledge that our measurement of the AT_MA_ was assessed using the tendon travel method, accounting for passive forces (44) at only one joint angle (i.e., at 90°). AT_MA_ is believed to change as function of joint angle (78–82), primarily as a result of calcaneal translation (83). However, when passive moments are correctly account for using the tendon travel method (44), or when AT_MA_ is determined from three-dimensional MR imaging (84), the AT_MA_ appears to remain constant across ankle angles. Despite these challenges, we did not see a significant group difference in ankle range of motion during the stance phase, nor differences in levels of muscle activation. If AT_MA_ does change with ankle angle and/or activation (84, 85), we presume these changes to be similar between groups. Importantly, previous studies examining the relationship between AT_MA_ and E_run_, where AT_MA_ was also measured at a single (passive) joint angle, which was the basis for our comparison of the previously determined AT_MA_ vs. E_run_ relationships. While the tendon travel method generally underestimates AT_MA_ compared to sagittal plane MR imaging when passive forces are not accounted for (50), all AT_MA_ were measured by the same investigator, using the same tendon travel method and AT_MA_ was corrected for passive forces (44). The AT_MA_ reported here are similar to those previously reported (44, 81). We also specifically compared short AT_MA_ to relatively longer AT_MA_ across participants, and so absolute AT_MA_ should not have impacted our results or their interpretations.

### Conclusion

The present study aimed to evaluate the relationship between AT_MA_ and E_run_ during submaximal running in recreationally trained runners. We have demonstrated that a longer AT_MA_ reduces the metabolic cost of triceps surae muscle contraction during stance. This reduction in muscle metabolic cost may translate to reductions in whole-body E_run_; however, we did not show a relationship between muscle metabolic cost and whole-body E_run_ nor did we test whether reductions in muscle metabolic cost directly translated to a reductions in E_run_ on a participant-by-participant basis, the latter of which would have required systematic changes to each participant’s AT_MA,_ and/or foot lever ratio, which was beyond the scope of this cross-sectional investigation.

By measuring muscle and tendon dynamics and energetics during running, we were able to, quantify the magnitude of AT energy storage and return and directly compare that with the estimated muscle metabolic cost during stance. In so doing, we have strengthened the notion that a low E_run_ may be accomplished not from storage and return of elastic energy itself, but by keeping muscle metabolic cost low. This notion is emphasized by our present results showing longer AT_MA_ are associated with a reduced muscle metabolic cost without a meaningful reduction in AT energy storage and return. These results also contribute to the direct measurement of potential explanations for short (or long) AT_MA_ contributing to a low E_run_, which until now have been largely theoretical.

## Data Availability

The raw data supporting the conclusions of this article will be made available by the authors, without undue reservation.
